# Epstein-Barr Virus Encephalitis as a Cause of Status Epilepticus – A Rare Encounter

**DOI:** 10.34172/aim.28844

**Published:** 2025-01-01

**Authors:** Ayisha Farooq Khan, Tooba Arshad, Faryal Abdy, Mohammad Wasay

**Affiliations:** ^1^Section of Neurology, Department of Medicine, Aga Khan University, National Stadium Road, Karachi, Pakistan

**Keywords:** Encephalitis, Epstein-Barr, Status epilepticus

## Abstract

Epstein-Barr virus (EBV) is associated with infectious mononucleosis and is known to cause various neurological complications, albeit rarely. EBV encephalitis is a rare manifestation, and even rarer is its presentation as status epilepticus. To date, fewer than ten cases of EBV encephalitis presenting as status epilepticus have been reported globally. We report a case of an 18-year-old girl who presented with fever followed by seizures. She went into status epilepticus the next day. Cerebrospinal fluid (CSF) analysis showed lymphocytic predominance. Brain imaging showed bilateral symmetric hyperintense lesions in the caudate and the putamen. EBV IgG and IgM were found to be positive. The patient was treated with ganciclovir and anti-epileptics and she improved gradually. Our case emphasizes the importance of considering EBV encephalitis as a potential cause of status epilepticus, even in individuals without immunodeficiency. Recognizing atypical presentations and appropriate diagnostic investigations can facilitate early diagnosis and treatment initiation.

## Introduction

 Epstein-Barr virus (EBV) is known to cause infectious mononucleosis. Patients are usually asymptomatic. Some may present with fatigue, fever, body aches, sore throat, pharyngitis, and lymphadenopathy. Fewer than 1% of patients with EBV develop neurological complications that include encephalitis, meningitis, acute disseminated encephalomyelitis, Guillain-Barre syndrome, transverse myelitis, or Bell’s palsy.^1,2^ Viral meningoencephalitis is the most common presentation. Uncommonly, akinetic mutism and status epilepticus may occur.^3^ Patients with EBV encephalitis may also have hepatomegaly or splenomegaly. But in cases where both might be absent, the diagnosis becomes a challenge.^4^

 The pathogenesis of EBV encephalitis is not well understood but may be secondary to direct infectious mechanisms and indirect immune reactions.^5^

 To the best of our knowledge, over the past two decades, there have been very few documented cases of EBV encephalitis presenting as status epilepticus that have been reported worldwide. Remarkably, none of these cases have been reported in Pakistan. Consequently, we present an inaugural report of EBV encephalitis in the Pakistani context. In this report, we describe a case of an immunocompetent young girl who went into status epilepticus and required intensive care unit treatment. She was diagnosed with EBV encephalitis and showed good recovery with ganciclovir. Our case not only contributes to the existing body of literature, but also emphasizes the importance of vigilance for unusual presentations of EBV-related neurological complications in immunocompetent individuals, particularly in regions where such cases are rare.

## Case Report

**Figure 1:**
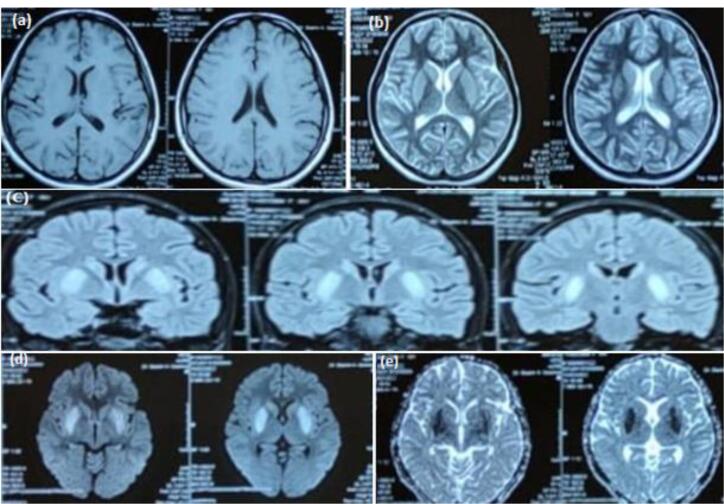
**(a)** T1 axial view showing hypo-intense signals in bilateral basal ganglia, T2 axial view **(b)** and FLAIR coronal view **(c)** showing hyper-intense signals in bilateral basal ganglia, DWI **(d)** showing diffusion restriction in the region of basal ganglia along with a signal drop out on ADC image **(e)**

 An 18-year-old female with no known comorbidities presented with fever for 15 days, drowsiness for 11 days and generalized tonic clonic seizures (GTCS) for 5 days. Initially, she was managed under the assumption of malaria, but her condition did not improve. She was later taken to another hospital when she experienced her first episode of GTCS, characterized by left-sided gaze, eye up rolling, and subsequent stiffening and jerking of all four limbs. This episode was followed by 15‒20 minutes of post-ictal amnesia. Thirty minutes later, she had another episode in the hospital, and intravenous (IV) administration of Levetiracetam 1 gm was initiated. MRI of the brain revealed bilateral basal ganglia hyperintensities (Figure 1).

 Although lumbar puncture was recommended, the family declined and brought the patient to our facility.

 The patient experienced continuous episodes characterized by a staring gaze, lip smacking, involuntary mouth movements, irrelevant talking, and excessive crying. These episodes lasted for 2‒3 minutes each and occurred back-to-back, without the patient fully regaining consciousness between them, resulting in an altered sensorium. An electroencephalogram (EEG) conducted 20 minutes later revealed continuous episodes of electrographic seizures. These seizures consisted of rhythmic, faster activity originating from the right frontal-central-temporal head region and spreading to the entire hemisphere, indicative of focal status epilepticus. Clinically, the patient exhibited involuntary mouth movements. In response, the dosage of intravenous levetiracetam was increased to 1g twice a day, and intravenous valproate was also increased to one gram twice a day.

 Despite these adjustments, the patient continued to experience similar episodes of lip smacking and altered sensorium. The antiepileptics were titrated to their maximum doses, and a third antiepileptic, lacosamide, was introduced following a loading dose. EEG was repeated which showed continuous multiple episodes of electrographical seizures arising from right temporo-occipital and then spreading to left hemisphere followed by 0.5‒1 second electro-decremental response. Each episode lasted for 50 seconds to one minute and was clinically associated with lip smacking. Lacosamide was increased to 200 mg twice a day and long lead EEG was attached. It continued showing multiple episodes of electrographical seizures.

 Since the electrographical seizures did not resolve even after 3 epileptics at maximal doses, and the patient was in status epilepticus, one gram methylprednisolone and an additional one gram of levetiracetam was given as stat doses and the family was counselled about the patient’s condition. The patient was electively intubated and shifted to the intensive care unit where midazolam and propofol infusion were started. The interval between the electrographical seizures increased on EEG along with a reduction in seizure frequency. Lumbar puncture was done. Cerebrospinal fluid (CSF) analysis showed a lymphocytic predominance, and EBV viral capsid IgG and IgM both were positive. As advised by the infectious disease specialist, cytomegalovirus polymerase chain reaction (CMV PCR) in CSF was also sent and meanwhile treatment with ganciclovir 250 mg twice a day was initiated, which was advised to continue for 3 weeks. The patient demonstrated clinical and electrographic improvement, leading to successful extubation. CMV PCR later came out to be negative. Her seizures also showed improvement, and she was eventually discharged home on lacosamide, levetiracetam, and valproate.

 At the one-month follow-up, the patient showed significant improvement. The frequency of her seizures had decreased markedly, and she had experienced only four episodes since being discharged home.

## Discussion

 Our case report highlights a rare presentation of EBV encephalitis, characterized by the occurrence of status epilepticus. While neurological complications of EBV infections are uncommon, they can have significant implications for patient management and prognosis. To date, only few cases of EBV infection presenting as status epilepticus have been reported in the literature. Table 1 provides a summary of these cases, along with our case.

**Table 1:** Summary of All the Cases

**Authors**	**Clinical Presentation**	**MRI Brain Findings**	**Treatment and Outcome**
Rodrigo-Armenteros et al^3^	14-year-old boy with akinetic mutism and non-convulsive status epilepticus	Bilateral swelling of basal ganglia	Improved on IV methylprednisolone
Mikuc et al^6^	10-year-old girl with fever for 6 days and status epilepticus	Cortical edema and subcortical hyperintensities in the T2 sequence.	Improved on intravenous acyclovir, intravenous immunoglobulins, and methylprednisolone.
Bains et al^7^	5-year-old boy with status epilepticus	Leptomeningeal thickening and T2-weighted/FLAIR hyperintense signal in bilateral caudate, putamen and hypothalamic regions.	Improved after 14 days of intravenous acyclovir and 5 days of high-dose methylprednisolone followed by oral prednisone.
Nishie et al^8^	37-year-female with headache and fever for 10 days. And now with status epilepticus	2‒3 mm lesion of the cerebellar white matter, which was suggestive of a small, demyelinated focus.	Improved
Glaser et al^9^	One patient had EBV- Details not known		
Greco et al^10^	3-year-old with confusion and partial motor seizures involving left arm and leg for 3 hours	Subcortical increased signal in the right occipital lobe	Improved on acyclovir.
DENİZ et al^11^	17-year-female with status epilepticus	Hyperintensity in the axial section of the flair sequence in the bilateral thalamic and parietooccipital regions	No improvement
Lehrnbecher et al^12^	Encephalitis, status epilepticus	Basal ganglia lesion	No effect with acyclovir. Improved after second course of steroids
Our case	18-year-old female with drowsiness and seizures	Bilateral basal ganglia hyperintensities	Improved on ganciclovir

 It is to note that most of the patients were children, and only one was an adult who suffered from EBV encephalitis and presented with status epilepticus. Most of these patients had involvement of basal ganglia and almost all of these patients showed good recovery and were discharged home, similar to our patient. Studies have demonstrated a link between EBV with N-methyl-D-aspartate (NMDA) encephalitis.^13^ Hence, in patients with persistent symptoms it is essential to send NMDA antibodies. Our patient, however, tested negative for these antibodies.

 The diagnosis of EBV encephalitis is typically made by detecting EBV antibodies or PCR in the blood or CSF. Detailed CSF reports of these patients show a viral picture. Head CT may be normal in the initial stages. Brain magnetic resonance imaging (MRI) findings may be non-specific and include the involvement of the cerebral hemisphere, basal ganglia, thalamus, brain stem, limbic system, cerebellum, and corpus callosum. In 40% of the patients, MRI may be normal.^14^ Diffusion restriction may also be seen in a few cases. Brain MRI in our patient showed involvement of the basal ganglia along with diffusion restriction.

 It is to note that although pathological crying is usually associated with cortical lesions, it may also occur in subcortical lesions involving the basal ganglia^15^ as seen in our patient.

 Interestingly, imaging may be also useful for prognostication. A case described by Tynell et al showed that the highest number of sequelae occurred in patients with thalamic or limbic system involvement, and the highest mortality was associated with brainstem involvement.^16^

 EBV encephalitis usually resolves on its own. Some studies have suggested that administration of acyclovir may reduce the duration of symptoms.^16^ Another study conducted in 2018 investigated the use of ganciclovir, valganciclovir, or valaciclovir in 48 patients diagnosed with EBV encephalitis. Among them, 26 patients fully recovered, 21 patients experienced disabling sequelae, and one patient was shifted to the intensive care unit because of a worsening condition. No deaths were reported.^17^ In our case, the patient was treated with ganciclovir, and she recovered. Unfortunately, repeat brain imaging could not be done as the patient was lost to follow-up.

 EBV encephalitis is often misdiagnosed in various parts of the world due to the unavailability of the CSF EBV PCR. And hence in that case, it is imperative to send EBV serology whenever there is evidence of EBV on the brain imaging and CSF reveals a viral picture.

 Our case report emphasizes the importance of considering EBV encephalitis as a potential etiology in patients presenting with status epilepticus, even in immunocompetent individuals. Recognizing the atypical manifestations of EBV infections and conducting appropriate diagnostic investigations, such as EBV serology or PCR, can aid in accurate diagnosis and timely initiation of treatment. Additionally, neuroimaging findings and their correlations with clinical outcomes can provide valuable prognostic information. Further research is warranted to enhance our understanding of the pathogenesis, optimal diagnostic approaches, and management strategies for EBV encephalitis, particularly in rare presentations such as status epilepticus.

 To conclude, EBV encephalitis presenting with status epilepticus is rare and can be seen in both immunocompetent and immunocompromised patients. EBV infection should always be kept in mind in patients with seizures or altered behavior, with typical brain imaging findings and a viral CSF picture. In such patients, it is strongly suggested that EBV serology or PCR be sent in blood or CSF and if the patient does not improve, then a trial of ganciclovir may be given. Follow-up brain imaging and CSF studies should also be done to check the viral load.
